# Fast quasi-null-filling of radiation patterns for multiple solutions generation

**DOI:** 10.1038/s41598-024-54497-9

**Published:** 2024-02-16

**Authors:** Cibrán López-Álvarez, María Elena López-Martín, Juan Antonio Rodríguez-González, Francisco José Ares-Pena

**Affiliations:** 1 Barcelona Research Center in Multiscale Science and Engineering, Polytechnic University of Catalonia, Campus Diagonal-Besòs, 08019 Barcelona, Spain; 2 Department of Physics, Polytechnic University of Catalonia, 08034 Barcelona, Spain; 3 Department of Morphological Sciences, University of Santiago de Compostela, 15782 Santiago de Compostela, Spain; 4 Department of Applied Physics, University of Santiago de Compostela, 15782 Santiago de Compostela, Spain

**Keywords:** Electrical and electronic engineering, Applied physics, Electrical and electronic engineering, Applied physics

## Abstract

Here we present an improved, rapid method for filling quasi-nulls in symmetrical radiation patterns synthesized by equispaced linear arrays, leading to the generation of multiple solutions. Considering the polynomial representation of the pattern, this null-filling is achieved by displacing the roots radially off the unit circle, keeping a constant displacement. This allows analyzing how the potential solutions vary with the quasi-uniform filling and the associated directivity loss. This method is based on the Cardano-Vieta relations, which link the coefficients of a complex Schelkunoff polynomial with its roots. As examples of application, we have considered a 20/100 element Dolph-Chebyshev pattern, with a spacing between the elements $\lambda / 2$, side lobe level of − 20/− 28 dB and three inner sidelobes at − 40/− 50 dB.

## Introduction

Sum patterns with equal side lobe levels tend to present array excitations with large excitation peaks (edge bightening) at the ends (this is, with a non-monotonic distribution). Such peaks indicate an increase in the tolerance sensitivity, apart from being disadvantageous in terms of implementation (an array whose aperture distribution is rapidly varying is difficult, or even impossible, to realize because of the mutual coupling) and susceptibility to edge effects^1^.

However, from shaped-beam synthesis, it is known that there are $2^M$ solutions for apertures with *M* filled-in nulls^1,2^. Such a multiplicity of solutions for the synthesis of the patterns leads to using an enumerative procedure for selecting the one with the most regular excitation. For the synthesis of sum patterns, by replacing the infinitely deep nulls by shallow nulls, a set of multiplicity of aperture distributions for the same pattern is then obtained. Among these solutions, it is possible to search that aperture that shows a minimal amplitude variability by minimizing the dynamic range ratio ($|I_{max} / I_{min}|$) or the maximum local smoothness ($|I_n / I_{n \pm 1}|_{max}$) at the expense of a small disminution of the pattern directivity. Previous studies have been synthesized sum patterns with filled nulls by means of the Orchard–Elliott method^3–5^ and found the optimal solutions by the use of genetic algorithms^6^.

Opposed to Orchard–Elliott method, our proposal is not actually a standard synthesis technique, as it needs as input a synthesized diagram that could be obtained by that method^5^. Concretely, we introduced a fast quasi-null-filling technique which quickly fills a radiation pattern, so that the solution is more easily implemented, starting from any non-filled diagram.

General non-linear optimization problems^7^ for real functions are based on finding the maximum/minimum of an objective function given some restrictions, defined by a series of equalities and inequalities. An interesting case is the convex programming problem. Concretely, an optimization problem is convex if, and only if, the objective function and the feasible region are convex. This is, the equality and inequality restrictions must be convex functions (such as affine or quadratic functions, or norms of vectors like the Euclidean norm, the absolute value, and the maximum value of a set of elements) in order for the feasible region to be convex as well. These inequalities have to be upper bounds; otherwise, lower bounds do not generally lead to convex functions^8^.

Convex optimization for antenna array patterns synthesis has been introduced by Lebret and Boyd^8^, which defined a constrained optimization problem of the pattern synthesis in some desired region also allowing the constrain of the beam level in other regions. Posteriorly, this technique has been highly employed in various applications by Isernia et al.^9–11^. For shaped beam synthesis, Fuchs, Skrivervik and Mosig^12^ pointed out that this technique can only be applied to uniformly spaced linear arrays composed of isotropic elements and conjugate symmetric excitations which are enforced on the array elements. For the latter, the far field radiated by the array is then a real function, and any lower/upper bound constraint on the radiated far field is therefore affine. The constraints and consequently the synthesis problem are then convex, although only upper-bound inequalities are known to be convex. Constraining the side lobe level in shaped beam diagrams leads to null-filling, associated to solutions whose excitations must produce real patterns. An extension of convex optimization (convex relaxation) was developed by Fuchs^13^. Bui et al.^14^ introduced a new technique which grants the maximum possible bandwidth given some side lobe level performance, considering the synthesis of equispaced linear arrays of isotropic elements. Such an strategy was validated with an array of realistic radiating elements fed with the excitations of equispaced linear array generated with the method. The null-filling effect can be also seen in these results.

According to Elliott^15^ and Kelley and Stutzman^16^, in very large arrays, the physical location of neighboring elements and the local distribution of excitations is almost the same for all the elements (except for those near the ends or the periphery). As a consequence, it is usually assumed that, in these large arrays, most of the excitations present similar active impedance (which depends on the array excitation and therefore vary with scan angle). Nevertheless, this active impedance is not the same as the self-impedance of an isolated element, even when the latter premise holds, and we need to consider mutual coupling to nearest neighbors. For small arrays, this assumption is not valid, as the active impedance may vary widely between elements. The same technique for determining active impedance in small arrays can be applied with equal success to the large array problem, since the ’common’ active impedance in large arrays is affected mostly by nearest neighbors, that is, by a small local array. As Isernia et al. argue^10,11^, it is not necessary to consider mutual coupling. Our approach can be seamlessly applied alongside the active element patterns proposed by Kelley and Stuzman^16^. This method enables the synthesis of microstrip array excitations without any approximation on the radiated field, surpassing all other synthesis techniques.

The main beam will scan if a controllable uniform progressive phase can be attached to the current distribution of an array which has been designed to produce a sum pattern^15^. As a result, pattern distortion and input impedance disturbance are introduced due to this scanning feature (the bigger the scan angles, the more severe these effects usually are). This is caused by changes in mutual coupling and electrical lengths of those segments of the feeding structure which contain the phase-shifters. Scanning issues become less critical with larger arrays, as mutual coupling tends to converge to a common value for all elements, and the coupling to the main line per element also decreases, given the higher number of elements. Nevertheless, it is important not to disregard these problems.

It would be desirable to develop a fast method of analyzing the influence of the null-filling level in the diminution of the aperture variability and its corresponding loss of directivity, in order to get a good compromise solution among both parameters. To the best of our knowledge, this is the first time that a method is able to achieve this without need of iteratively solving a set of matrix equations for each null-filling level, as the Orchard–Elliott method^5^ would require. As examples of application, we have considered two linear array that synthesize Dolph–Chebyshev patterns with reduced inner side lobes.

## Results

**Figure 1:**
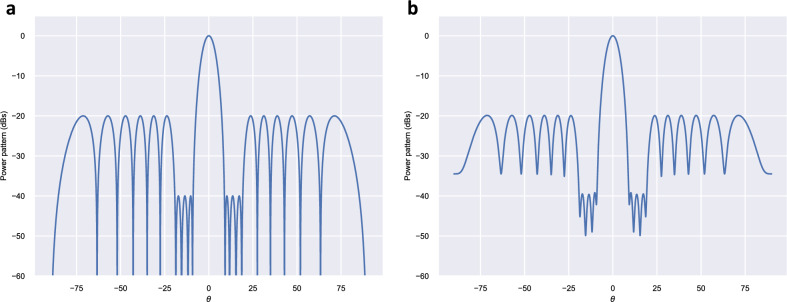
20-element Dolph-Chebyshev pattern, with a spacing between the elements $d = \lambda / 2$, side lobe level $SLL = -\,20 ~\text {dB}$ but with three inner sidelobes at − 40 dB, presenting (**a**) the unfilled pattern ($a_r = 0.0000$), obtained by the Orchard-Elliott method, and (**b**) the previous pattern after applying a quasi-null-filling corresponding to $a_r = \pm \,0.0200$.

Here we apply the previously introduced methodology to a 20-element Dolph–Chebyshev pattern, with a spacing between the elements of $d = \lambda / 2$, side lobe level $SLL = -\,20 ~\text {dB}$ (the optimal sidelobe level that maximizes the directivity of a 20-element Dolph-Chebyshev pattern) but with three inner sidelobes at $-\,40 ~\text {dB}$. The initial (unfilled) pattern, obtained by using the Orchard–Elliott method^5^ is shown in Fig. 1a.

**Figure 2:**
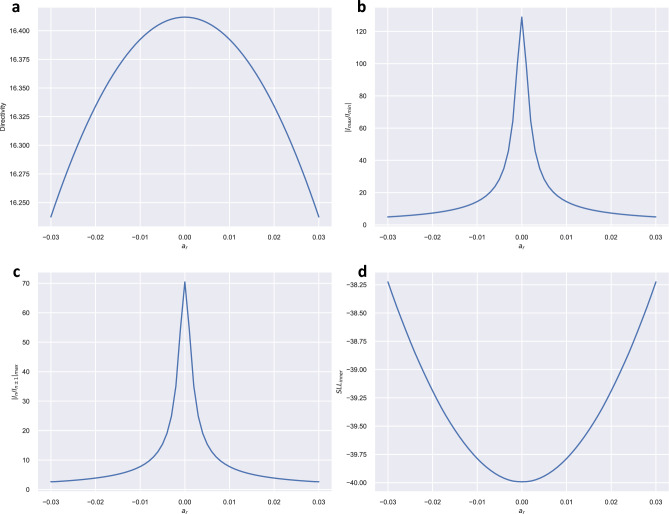
(**a**) The directivity shows a bowing effect in terms of $a_r$, with a maximum of $D = 16.41$ at $a_r = 0$, (**b**) the absolute value of maximum intensity normalized to its minimum value ($|I_{max} / I_{min}|$) has a peak of 128.87 at $a_r = 0$, (**c**) the absolute value of the maximum ratio of the intensity with respect to the previous or next intensity present a peak of 70.45 at $a_r = 0$, (**d**) the SLL shows a bowing effect in terms of $a_r$, with a minimum of $-\,40.00$ dB at $a_r = 0$, while it rapidly increases for other $a_r$ values.

We applied the proposed method considering values of $a_r$ in the interval [− 0.0300, 0.0300]. For each value, the best solution minimizing $|I_{max} / I_{min}|$ or $|I_n / I_{n \pm 1}|_{max}$ was obtained by searching among $2^{19}$ possible distributions generating the same power pattern. The value of directivity and the sidelobe level of the inner lobes for each $a_r$ value was also obtained ($SLL_{inner}$). Figure 2 shows the dependence of these parameters with $a_r$. Table 1 summarizes the values of these parameters for $a_r = \{0.0000, \pm \,0.0100, \pm \,0.0200, \pm \,0.0300\}$. Comparing with the unfilled pattern, we have found that for $a_r = 0.0200$, the proposed method allowed a reduction of 94.3% of the $|I_{max} / I_{min}|$ parameter (from 128.87 to 7.28), a diminution of $|I_n / I_{n \pm 1}|_{max}$ of 94.5% (from 70.45 to 4.89), at the expense of a loss of directivity of only 0.5% (from 16.41 to 16.33) and an increase of 0.8 dB of the inner side lobes. Figure 1b, shows the resulting quasi-null-filled pattern for this case whereas Table 2 shows the corresponding roots (including the necessary $a_r$ signs to get the optimal solution) that synthesize this pattern as well as the excitations of the array elements.

**Table 1:** Different values of directivity, variability of excitations and SLL for different values of $a_r$ (Fig. 1b).

	Filling level			
	$a_r = 0.0000$	$a_r =\pm \,0.0100$	$a_r = \pm \,0.0200$	$a_r = \pm \,0.0300$
Directivity	16.41	16.39	16.33	16.24
$|I_{max} / I_{min}|$	128.87	14.39	7.28	4.90
$|I_n / I_{n \pm 1}|_{max}$	70.45	7.78	3.89	2.64
$SLL_{inner}$	$-\,40.0 ~\text {dB}$	$-\,39.7 ~\text {dB}$	$-\,39.2 ~\text {dB}$	$-\,38.2 ~\text {dB}$

**Table 2:** Different values of $a_r, b_n$, amplitude and phase depending on *n* (Fig. 3b).

	Roots $e^{a_r + j b_n}$		Relative excitations	
*n*	$a_r$	$b_n$ (rad)	Amplitude	Phase (rad)
1	$-\,0.0200$	$-\,2.8077$	0.9802	0.0004
2	$-\,0.0200$	$-\,2.4734$	0.2520	$-\,88.7063$
3	$-\,0.0200$	$-\,2.1379$	0.4270	$-\,5.4635$
4	$-\,0.0200$	$-\,1.7995$	0.9744	$-\,13.3906$
5	$-\,0.0200$	$-\,1.4519$	1.1554	$-\,13.5009$
6	$-\,0.0200$	$-\,1.0061$	1.3038	$-\,16.7949$
7	$-\,0.0200$	$-\,0.8365$	1.3030	$-\,18.2566$
8	$-\,0.0200$	$-\,0.6413$	1.4896	$-\,17.9819$
9	$-\,0.0200$	$-\,0.5042$	1.6444	$-\,17.8238$
10	0.0200	0.5042	1.8357	$-\,16.5435$
11	0.0200	0.6413	1.8013	$-\,17.4130$
12	0.0200	0.8365	1.6818	$-\,16.8577$
13	0.0200	1.0061	1.4567	$-\,19.0758$
14	0.0200	1.4519	1.3394	$-\,17.0779$
15	0.0200	1.7995	1.2680	$-\,18.0038$
16	0.0200	2.1379	1.1953	$-\,12.4146$
17	0.0200	2.4734	0.9392	$-\,14.7440$
18	0.0200	2.8077	0.4752	$-\,3.7653$
19	$-0.0200$	3.1416	0.2593	262.4217
20	–	–	1.0000	0.0000

**Figure 3:**
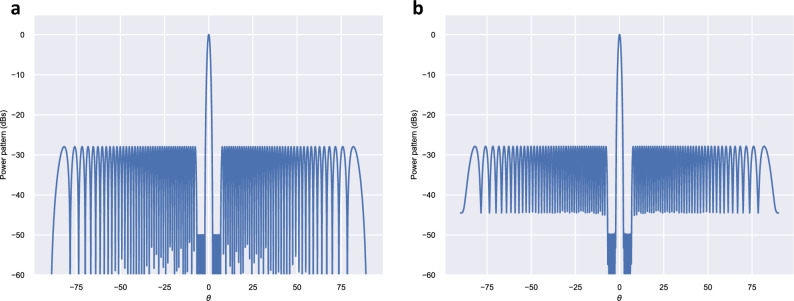
20-element Dolph–Chebyshev pattern, with a spacing between the elements $d = \lambda / 2$, side lobe level $SLL = -\,28 ~\text {dB}$ but with six inner sidelobes at − 50 dB, presenting (**a**) the unfilled pattern ($a_r = 0.0000$), obtained by the Orchard-Elliott method, and (**b**) the previous pattern after applying a quasi-null-filling corresponding to $a_r = \pm \,0.0030$.

In order to extend our procedure to a larger array, we have considered a 100-element Dolph–Chebyshev pattern, with a spacing elements of $d = \lambda / 2$, side lobe level $SLL = -\,28 ~\text {dB}$ (the optimal sidelobe level that maximizes the directivity of a 100-element Dolph-Chebyshev pattern) but with six inner sidelobes at $-\,50 ~\text {dB}$. The initial (unfilled) pattern obtained using the Orchard Elliott method^5^ is shown if Fig. 3a.

**Figure 4:**
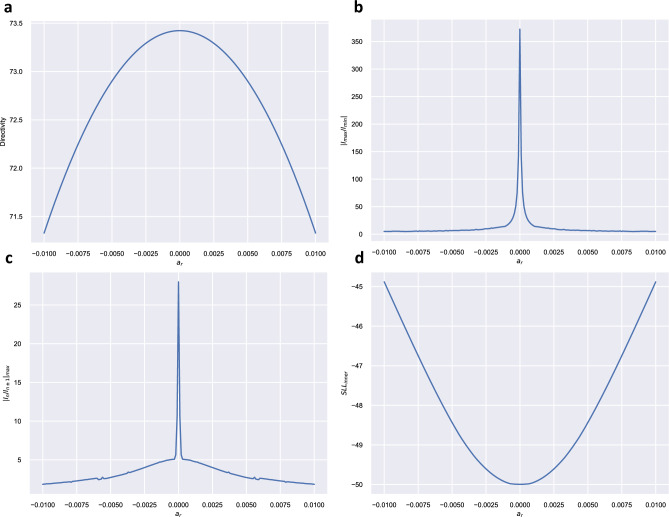
(**a**) The directivity shows a bowing effect in terms of $a_r$, with a maximum of $D = 73.42$ at $a_r = 0$, (**b**) the absolute value of maximum intensity normalized to its minimum value ($|I_{max} / I_{min}|$) has a peak of 372.55 at $a_r = 0$, (**c**) the absolute value of the maximum ratio of the intensity with respect to the previous or next intensity present a peak of 27.99 at $a_r = 0$, (**d**) the SLL shows a bowing effect in terms of $a_r$, with a minimum of $-\,50.00$ dB at $a_r = 0$, while it rapidly increases for other $a_r$ values.

In this case, we applied the proposed method considering values of $a_r$ in the interval [− 0.0100, 0.0100]. For each value, the best solution minimizing $|I_{max} / I_{min}|$ or $|I_n / I_{n \pm 1}|_{max}$ was obtained by using a genetic algorithm^17^ to search among $2^{99}$ possible solutions generating the same power pattern. Calculations were performed using the SUGAL library^18^. The number of chromosomes in the population (population size) was 500 (each chromosome has 99 genes/bits). In each generation, a ranked replacement of parents bettered by their offspring was performed. Offspring were generated by one point crossover and the inclusion of one mutation on every chromosome. The final solution was obtained after 1000 generations. We also tried to run the genetic algorithm using a larger population but we have not found appreciable improvements. As in the previous example, we have also calculated the directivity and the sidelobe level of the inner lobes for each $a_r$. Figure 4 shows the dependence of these parameters with $a_r$. Table 3 summarizes the values of these parameters for $a_r = \{0.0000, \pm \,0.0010, \pm \,0.0030, \pm \,0.0050, \pm \,0.0070, \pm \,0.0100\}$. Comparing with the unfilled pattern, we have found that for $a_r = 0.0030$, the proposed method allowed a reduction of 97.8% of the $|I_{max} / I_{min}|$ parameter (from 372.55 to 8.12), a diminution of $|I_n / I_{n \pm 1}|_{max}$ of 86.8% (from 27.99 to 3.69), at the expense of a loss of directivity of only 0.25% (from 73.42 to 73.23) and an increase of $0.8 ~\text {dB}$ of the inner side lobes. Figure 3b, shows the resulting quasi-null-filled pattern for this case. It is remarkable that due to the exponential growth of the number of solutions, the improvement achieved with the proposed method is greater in larger arrays: for the 100-element array applying a small filling level ($a_r = 0.0010$) allows to find solutions that show great improvements on $|I_{max} / I_{min}|$ and $|I_n / I_{n \pm 1}|_{max}$ (of 95.9% and 82.6%, respectively) keeping the directivity and SLL of the pattern practically unchanged.

As shown in Table 2, we considered an even number of elements, thus the optimal solution is complex-asymmetric^19^ (useful in end-fed arrays). If we were interested in a complex-symmetric optimal solution, we would need an odd number of element (useful in center-fed arrays).

**Table 3:** Different values of directivity, variability of excitations and SLL for different values of $a_r$ (Fig. 3b).

	Filling level					
	$a_r = 0.0000$	$a_r =\pm \,0.0010$	$a_r = \pm \,0.0030$	$a_r = \pm \,0.0050$	$a_r = \pm \,0.0070$	$a_r = \pm \,0.0100$
Directivity	73.42	73.40	73.23	72.90	72.40	71.32
$|I_{max} / I_{min}|$	372.55	15.34	8.12	6.03	5.53	4.89
$|I_n / I_{n \pm 1}|_{max}$	27.99	4.86	3.69	2.71	2.39	1.83
$SLL_{inner}$	$-\,50.0 ~\text {dB}$	$-\,39.7 ~\text {dB}$	$-\,39.2 ~\text {dB}$	$-\,48.3 ~\text {dB}$	$-\,47.11 ~\text {dB}$	$-\,44.89 ~\text {dB}$

## Discussion

A novel approach that allows performing fast quasi-uniform null filling in sum patterns synthesized by equispaced linear arrays has been described. This procedure allows to obtain aperture distributions that show an amplitude variability much smaller than the obtained with the unfilled pattern at the expense of a minimum loss of directivity and increase in the level of the inner sidelobes. This method is directly applicable to asymmetric patterns, to difference patterns, and even to shaped beams, as well as to linear and circular Taylor distributions by introducing complex roots with a constant imaginary part that will produce the required quasi-null-filling. Based on the principle of collapsed distributions^15^, it is also possible to apply this approach to planar arrays.

## Methods

As it is well known, a linear array of $N+1$ radiating elements which lay out in a equally-spaced grid (with intervals *d*) along the z-axis presents the array factor^15^:

1 $$\begin{aligned} F(w) = \sum _{n=0}^N I_n w^n = I_N \prod _{n=1}^N \left( w - w_n \right) \end{aligned}$$

being $I_n$ the relative complex excitation for the *n*-th element and $w = e^{j \psi }$ the roots of the Schelkunoff polynomial ($\psi = k d \cos {\theta }$, with *k* the wavenumber and $\theta$ the angle from endfire). Concretely, any linear array synthesis problem can be understood as finding the optimal positions of roots $w_n$; for that end, the Schelkunoff *w* plane can be used as a design tool. Given *M* roots $w_n$ lying out of the unit circle, so that *F*(*w*) is nonzero (”null filled”) in the corresponding directions, then there are $2^M$ different sets of excitations $I_n$ which can be considered ”power equivalent” in the sense that they give (to within a constant factor) the same power pattern $F(w) F(w)^*$ (* denotes complex conjugate). Excitations which belong to this set of solutions are derived from each other by root replacement $w_n = e^{a_n + j b_n}$ by $w'_n = e^{- a_n + j b_n}$. It has been reported a genetic algorithm method for optimizing these $2^M$ equivalent distributions of excitations, finding the most suitable one, this is, the one that minimized $|I_{max} / I_{min}|$ and/or $|I_n / I_{n \pm 1}|_{max}$^6^.

By computing the productory $\prod _{n=1}^N \left( w - w_n \right)$ and applying the principle of identity (two polynomia which have identical numerical values for a number of *w* values greater than the degree of both, then they are identical, this is, they have the same degree and coefficients), we get to the fundamental relationships between roots and coefficients:

2 $$\begin{aligned} \begin{gathered} - \frac{I_{N-1}}{I_N} = \sum _i w_i \\ \frac{I_{N-2}}{I_N} = \sum _{i,j} w_i w_j \\ - \frac{I_{N-3}}{I_N} = \sum _{i,j,k} w_i w_j w_k \\ \dots \\ (-1)^N \frac{I_0}{I_N} = \prod _i w_i \end{gathered} \end{aligned}$$

which are known as Cardano–Vieta relations (that establishes the relation among the polynomial roots and its coefficients).

Consider all roots to have the same amplitude, this is, $w_n = e^{a_r} e^{j b_n}$, which gives:

3 $$\begin{aligned} \begin{gathered} - \frac{I_{N-1}}{I_N} = e^{a_r} \sum _i e^{j b_i} \\ \frac{I_{N-2}}{I_N} = e^{2 a_r} \sum _{i,j} e^{j b_i} e^{j b_j} \\ - \frac{I_{N-3}}{I_N} = e^{3 a_r} \sum _{i,j,k} e^{j b_i} e^{j b_j} e^{j b_k} \\ \dots \\ (-1)^N \frac{I_0}{I_N} = e^{N a_r} \prod _i e^{j b_i} \end{gathered} \end{aligned}$$

so that depending on the value of $a_r$ we obtain a quasi-uniform null filling. Equation (3) shows that it is possible to change from a pattern with deep nulls to a pattern with quasi-uniform null filling just by multiplying the initial excitations by the factor $e^{n \cdot a_r}$, i.e.,

4 $$\begin{aligned} I_n^f = I_n^{uf} e^{n \cdot a_r} \end{aligned}$$

where $I_n^f$ and $I_n^{uf}$ are the excitations of the filled and unfilled pattern, respectively.

## Data Availability

The datasets used and/or analysed during this study are available from the corresponding author upon reasonable request.
